# Preparation and Unique Three-Dimensional Self-Assembly Property of Starfish Ferritin

**DOI:** 10.3390/foods12213903

**Published:** 2023-10-25

**Authors:** Chenxi Zhang, Xuemin Chen, Bo Liu, Jiachen Zang, Tuo Zhang, Guanghua Zhao

**Affiliations:** College of Food Science & Nutritional Engineering, China Agricultural University, Beijing 100083, China; zhangchenxi9556@163.com (C.Z.); s20213061072@cau.edu.cn (X.C.); s20193060993@cau.edu.cn (B.L.); zangjiachen@cau.edu.cn (J.Z.); zhangtuo@cau.edu.cn (T.Z.)

**Keywords:** *Asterias forbesii* ferritin, π–π interactions, two 3D nanocage architectures, interconversion

## Abstract

The structure and assembly properties of ferritin derived from aquatic products remain to be explored. Constructing diverse three-dimensional (3D) protein architectures with the same building blocks has important implications for nutrient delivery, medicine and materials science. Herein, ferritin from *Asterias forbesii* (AfFer) was prepared, and its crystal structure was resolved at 1.91 Å for the first time. Notably, different from the crystal structure of other reported ferritin, AfFer exhibited a BCT lattice arrangement in its crystals. Bioinspired by the crystal structure of AfFer, we described an effective approach for manufacturing 3D porous, crystalline nanoarchitectures by redesigning the shared protein interface involved in different 3D protein arrays. Based on this strategy, two 3D superlattices of body-centered tetragonal and simple cubicwere constructed with ferritin molecules as the building blocks. This study provided a potentially generalizable strategy for constructing different 3D protein-based crystalline biomaterials with the same building blocks.

## 1. Introduction

Iron is an essential mineral element for human health that participates in multiple biological processes, including the tricarboxylic acid cycle, DNA synthesis and respiratory chain transmission [1]. Excessive free iron induces oxidative stress through the Fenton reaction, leading to ferroptosis, thereby contributing to the pathophysiology of diseases [2]. Ferritin is the main iron storage protein in most living organisms, playing a key role in iron homeostasis and detoxification [3]. Canonical ferritin is composed of 24 subunits, which self-assemble into a quasi-spherical nanocage with an outer diameter of ~12 nm and an interior diameter of ~8 nm. Ferritin has prominent octahedral 4-3-2 symmetry (Figure S1), and each ferritin molecule contains 24 *C*_3_–*C*_4_ interfaces, 12 *C*_2_ interfaces, 8 *C*_3_ interfaces, and 6 *C*_4_ interfaces, which provide several potential pathways for metal ions and small organic molecules to diffuse into its inner cavity. In addition to iron, ferritin exhibits a remarkable affinity for some nonferrous metal ions and metal-containing compounds, such as Ca^2+^, CuS, ferrocene derivatives, and cisplatin. Except for biomineralization, ferritin has the property of reversible self-assembly, with the nanocage being disassociated into subunits at an extremely acid/alkaline pH (≤2.0 or ≥11.0) and the resulting subunits reconstituting into a cage-like structure under neutral conditions. By taking advantage of this feature, multifarious food nutrients, therapeutic drugs, and contrast agents have been encapsulated within the inner cavity of ferritin to improve their stability and realize targeted delivery [4,5]. To sum up, due to the adequate structural features, unique peculiarity, high stability, and favorable biocompatibility, ferritin has been widely exploited as nanoscale reaction chambers, bioimaging agents, and delivery vehicles. So far, a number of studies on ferritin have focused on terrestrial animal and plant ferritin, while aquatic ferritin has been largely unexplored.

Like ferritin, many natural protein architectures are precise assemblies of protein subunits that can be viewed as molecular Lego sets, such as actin filaments, viral capsids, lumazine synthase, and carboxysome shell [6,7]. Subunit self-assembly facilitates the generation of elaborate systems of more advanced functionalities with high precision, efficiency, and adaptability. Moreover, food proteins such as α-lactalbumin, lysozyme and soyabean protein could self-assemble to form supramolecular architectures under specific conditions, sequentially being applied in nutrient delivery and food system stability [8]. Inspired by subunit self-assembly and natural protein assemblies, scientists endeavor to create artificial protein assemblies (from nm to μm), including one-dimensional (1D) nanofilaments or nanotubes [9,10], two-dimensional (2D) nanosheets [11,12] and three-dimensional (3D) protein crystalline arrays [13,14], that execute complex, multistep biochemical processes or form structural materials [15,16]. Here, 3D protein nanocage lattices have aroused considerable interest since the protein cavity can be filled with inorganic nanoparticles or proteins, thus producing highly ordered functional superlattice, e.g., 3D quantum dot organizations or enzymatic arrays [17,18,19,20]. Additionally, 3D protein nanocage lattices contain two spatially segregated compartments, namely the inherent inner cavity of the protein nanocage and the interglobular space of the lattice. Consequently, our recently constructed 3D *Thermotoga maritima* nanocage superlattices could serve as a platform for the hierarchical encapsulation of two different cargoes, Mg^2+^ and carbonic anhydrase for the efficient conversion of CO_2_ into MgCO_3_ nanoparticles under mild conditions [21,22], which represent a new class of protein vehicles for multicomponent encapsulation and delivery. Different 3D superlattices have distinct arrangement, interglobular spaces and emergent properties for use in the fields of food, nutrition, and medicine. However, constructing 3D protein lattices with a reversible structure transformation property remains a daunting task.

**Scheme 1:**
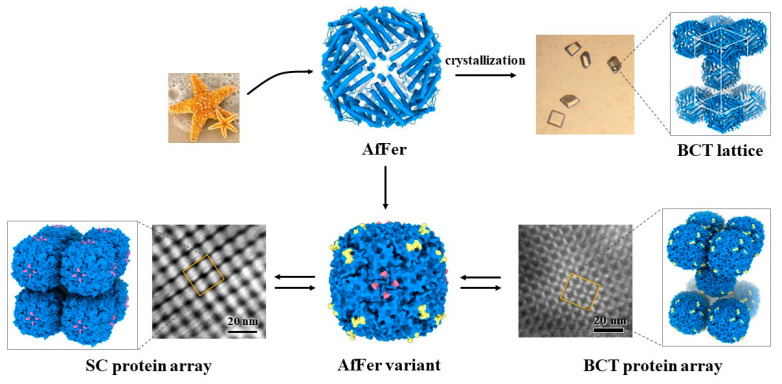
Schematic representation of the construction and interconversion of BCT and SC ferritin arrays. The Glu10, Asn9, and Lys120 of each ferritin subunit near its *C*_3_ interfaces are highlighted in yellow, and the His/Phe mutation around the *C*_4_ interfaces of ferritin are highlighted in pink.

In principle, the octahedral (432) symmetry of ferritin nanocages provides access to four different lattice arrangements, including simple cubic (SC) [14], face-centered cubic (FCC) [23], body-centered cubic (BCC), and body-centered tetragonal (BCT) arrangements [24,25]. Considering that *Echinodermata* is a phylum endemic to aquatic products, herein, ferritin from *Asterias forbesii* (AfFer) was first prepared. Subsequently, its crystal structure was resolved for the first time at 1.91 Å, and it exhibited a 24-meric nanocage structure and BCT lattice arrangement. Thereafter, we introduced a self-assembling strategy by designing π–π stacking interactions at the shared protein interfaces involved in two different kinds of 3D protein nanocage superlattices, which is referred to as key protein interface redesign (KPIR). As a proof-of-concept study, we introduced His/Phe at the *C*_4_ interface of AfFer and created 3D protein nanocage arrays with two different lattice arrangements, including SC and BCT. As expected, crystallographic analysis revealed that the designed residue sites are involved in interactions between the proteins in the two 3D lattices. Consequently, their reversible transformation was achieved using discrete ferritin nanocages as intermediates in response to different solution conditions, as shown in Scheme 1.

## 2. Materials and Methods

### 2.1. Protein Preparation and Purification

The mRNA sequence of *Asterias forbesii* ferritin (AfFer) was obtained from the National Center for Biotechnology Information (https://www.ncbi.nlm.nih.gov/search/all/?term=AF001984.1 accessed on 22 October 2023), and the GenBank number is AF001984.1 [26]. It was introduced into the restriction endonuclease cleavage sites of NdeI and BamHI in the expression vector PET-3a. The plasmid was transformed into *Escherichia coli* BL21 (DE3) competent cells, and the transformants were grown to an OD_600_ of 0.6 in 500 mL of LB medium containing 100 mg/L Amp at 37 °C. Then, 200 µM IPTG was add to induce protein expression, with further incubation at 16 °C for 12.0 h. The cells were harvested by means of centrifugation at 5000 rpm for 10 min and then resuspended in 50 mM Tris–HCl (pH 9.5) buffer. After ultrasonication for 15 min, the cells were incubated at 60 °C for 15 min, and then 60% ammonium sulfate was added to the supernatant after centrifugation. Following salting out for 6.0 h, the pellet was resuspended in 50 mM Tris−HCl (pH 9.5) and dialyzed against the above buffer 3 times. The supernatant was further purified via DEAE-Sepharose ion-exchange column chromatography with a 0–1.0 M NaCl gradient elution procedure and size-exclusion chromatography successively. The protein purity was confirmed by native-PAGE and SDS-PAGE (polyacrylamide gel electrophoresis). The protein concentrations were determined using a BCA protein assay kit.

AfFer plasmid DNA was extracted using a MolPure^®^ Plasmid Mini Kit from Yeasen Biotechnology Co., Ltd. (Shanghai, China). Site-directed mutagenesis was performed via PCR (polymerase chain reaction) with the AfFer gene as the template DNA, and the primers used in this process are listed in Table S1. The PCR conditions consisted of 1.0 µL (100 ng/µL) template DNA, 1.0 µL of each primer, 5.0 µL of 10 × PCR buffer for KOD-plus, 5.0 µL of 2.0 mM dNTP, 2.0 µL of 25.0 mM MgSO_4_, 1.0 µL of KOD-plus polymerase and 34.0 µL ddH_2_O in a final volume of 50.0 µL in a thermal cycler. PCR was initiated with an incubation step at 96 °C for 2 min, followed by 25 cycles of 94 °C/15 s, 60 °C/30 s, and 68 °C/6 min, with a final extension step at 68 °C for 10 min. The PCR products were digested with *Dpn* I enzyme for 1.0 h at 37 °C and then transformed into *Trelief*^TM^ 5α competent cells for the propagation of plasmid constructs. Ampicillin-resistant colonies were selected for the plasmid extraction, and the mutant plasmids were confirmed by DNA sequencing. The expression and purification of variants were the same as described for AfFer.

### 2.2. Protein Crystallization, Data Collection, and Structure Determination

Crystallization of AfFer and its mutants was performed with reference to other ferritins [27]. First, the purified ferritin was dissolved in 5.0 mM Tris–HCl buffer and concentrated to 12.0 µM. Secondly, Crystal Screen 1 and 2 and WIZARD Classic 1 and 2 kits from Hampton Research were used to screen the crystallization conditions. Here, 1.0 µL of protein was mixed with an equal volume of crystallization solution and equilibrated against 50 µL of reservoir solution at 20 °C. Thereafter, the crystals were optimized by means of hanging drop vapor diffusion using a 24-well plate under the conditions shown in Table S2. X-ray diffraction data were collected on the BL17U1 or BL18U beamlines of the Shanghai Synchrotron Radiation Facility (SSRF) and processed with HKL-3000 software (https://hkl-xray.com/hkl-3000) [28]. The structure of AfFer was resolved via the Phaser program of PHENIX1.18.2-3874 software using *Azumapecten farreri* ferritin (PDB code: 7VT2) as the searching model (AfFer was used as the searching model when resolving the AfFer mutant structures) [27]. The following refinement and manual rebuilding were carried out using PHENIX and COOT, respectively [29,30]. The data collection and refinement statistics are summarized in Tables S3–S5. All the figures of the protein structures were produced using ChimeraX 1.2.5 software [31].

### 2.3. Transmission Electron Microscopy (TEM) Analyses

Transmission electron microscopy images of the protein samples were observed and recorded using an H-7560B Hitachi TEM system operating at 80 kV. The protein samples were prepared as follows: 7.0 µL of protein solution was placed on a carbon-coated copper grid for 5 min, and then the excess solution was carefully removed along the edge of the copper grid with filter paper. After staining using 2% uranyl acetate for 5 min, the excess solution was also wicked away, and the copper grid was subsequently evaporated at room temperature for 20 min.

### 2.4. Small Angle X-ray Scattering (SAXS)

The SAXS data were recorded with a Xeuss2.0 SAXS/WAXS system (Cu Kα radiation, λ = 1.54 Å) in a *q* range from 0.042–2.21 nm^−1^ at room temperature (Xenocs, France) [14]. The distance between the sample and the detector was 2.50 m, and a silver behenate standard sample was used to calibrate the length of the scattering vector *q*. The samples were prepared by collecting the assemblies and sealing them in metal rings with polyimide tape. The two-dimensional scattering spectrum was recorded by exposing the sample for 240–480 s. The 1D scattering data were determined via the azimuthal averaging of the 2D data to obtain plots of the scattering intensity versus the scattering vector *q*:

(1) q=4πsin θ/λ

where *θ* is one half of the scattering angle and λ is the X-ray wavelength. The diffraction peaks were compared with the permitted reflections of different periodic structures to determine the Bravais lattice type in each sample. The interplanar distance of the cubic crystal system conforms to the equation:

(2) $$1/d^{2}=(h^{2}+k^{2}+l^{2})/a^{2}$$

The interplanar distance of the tetragonal crystal system conforms to the equation:

(3) $$1/d^{2}=(h^{2}+k^{2})/a^{2}+l^{2}/c^{2}$$

where *h*, *k*, and *l* are the Miller indices, while *a* and *c* are the unit cell parameters. For the Bravais lattice in this article, the unit cell parameter *a* of the simple cubic and body-centered tetragonal crystal systems is consistent with the theoretical outer diameter (R) of the ferritin molecule.

## 3. Results and Discussion

### 3.1. Crystal Structure of Asterias Forbesii Ferritin (AfFer)

**Figure 1:**
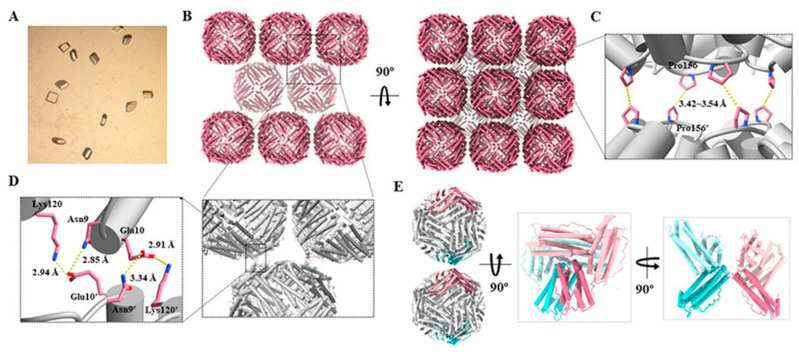
X-ray diffraction (PDB ID: 8IQV) characterization of AfFer. (**A**) Optical microscope image of AfFer crystals at pH 8.0, 15% (*v*/*v*) ethanol, and 200 mM MgCl_2_. (**B**) Side and top views of the 3D AfFer arrays in the crystal structure. (**C**) Close-up view of the proline residues at the *C*_4_ interface between two adjacent AfFer molecules. (**D**) Close-up view of the electrostatic interactions between two adjacent AfFer molecules along the *C*_3_ interface. (**E**) The *C*_3_ interfaces directly involved in AfFer’s intermolecular interactions. The subunits involved in electrostatic interaction are highlighted as dark pink and dark turquoise, and other subunits constituting the *C*_3_ interface are highlighted as pale pink and turquoise.

The preparation and purification of AfFer was performed as we have reported in relation to other ferritins, with some modifications [27]. According to the native-PAGE and SDS-PAGE shown in Figure S2, AfFer has been purified to homogeneity. The protein subunit contains 171 amino acids and has a molecular weight of about 19.36 kDa. The electrophoresis results are in good agreement with this theoretical calculation, and the protein consists of about 24 subunits. In order to clarify the detailed structural features of AfFer, the crystal structure was subsequently solved at a resolution of 1.91 Å for the first time. The space group is *I422*, with unit-cell parameters of a = b = 116.52 Å, c = 205.62 Å, and *α* = *β* = *γ*. These lattice parameters indicate that starfish ferritin is aligned along eight *C*_3_ interfaces (which can be approximated as the eight vertices of a cube) to form a body-centered tetragonal (BCT) lattice in the crystal (Figure 1A,B, Tables S2 and S3, PDB ID: 8IQV).

The AfFer molecule is located in the center of the hexahedron contacting with the other eight ferritin molecules located at the vertices of the hexahedron through electrostatic interactions mediated by Glu10-Asn9’/Lys120’ (Figure 1D). The *C*_4_ interface of this ferritin molecule forming the subface is perfectly aligned by virtue of Pro156, with weak van der Waals forces that provide assistance for the stability of the BCT lattice (Figure 1C). Since only one out of three subunits constituting the *C*_3_ interface is involved in the formation of electrostatic interactions, the dislocation between the *C*_3_ interfaces of the ferritin molecules results in the lack of crystallographic three-fold symmetry (Figure 1E); consequently, AfFer forms BCT rather than a BCC lattice. Notably, this BCT lattice differs from most reported ferritin molecules, which are packed in a FCC fashion along the *C*_2_ interface in their crystals. Also, the *C*_4_ interfaces of the AfFer forming the subface are close together, unlike the very few reported examples of BCT formed in horse spleen ferritin crystals or ferritin–MOFs.

### 3.2. Design of the Two 3D Protein Nanocage Superlattices

Based on the distinctive BCT arrangement of the AfFer nanocages, and the fact that the octahedral symmetry of the assembled 24-meric AfFer cage endows it with three *C*_4_ rotation axes that are perpendicular to each other, it was found that the intermolecular interactions at the *C*_4_ interface participate in the construction of both BCT and SC lattices. Therefore, we envisioned that if we strengthened the forces at the protein *C*_4_ interface while keeping the inherent driving forces located at the *C*_3_ interfaces responsible for the formation of the BCT lattice, the AfFer molecules would form two types of 3D lattices (SC and BCT) separately, depending on the solution conditions. To confirm this idea, we planned to build π–π interactions at the *C*_4_ interface. Upon analysis of the crystal structure of AfFer, we deemed Pro156 an ideal position for anchoring the His motif, because the side chain of Pro156 located on the exterior of the *C*_4_ interface that protrudes on the outside surface of the protein cage (Figure S3). Subsequently, Pro156 was replaced with His, termed ^P156H^AfFer. This mutant was purified to homogeneity, as suggested by its native-PAGE and SDS-PAGE results (Figure S4).

### 3.3. Construction and Characterization of the BCT Superlattices

**Figure 2:**
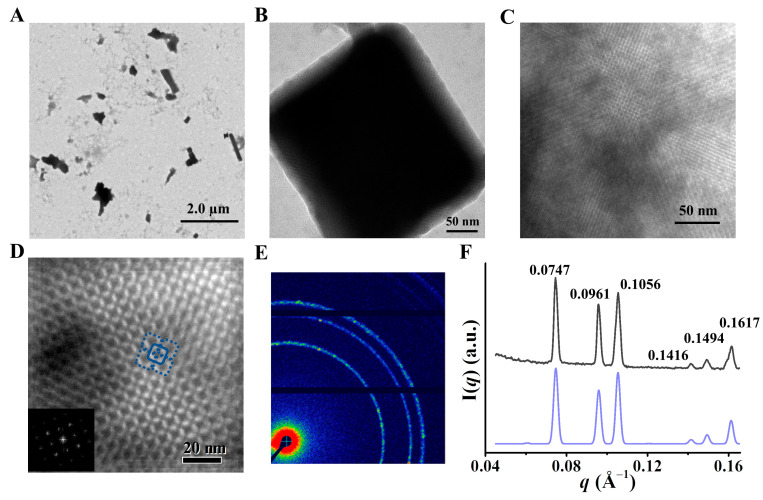
Characterization of the ^P156H^AfFer assemblies at pH 8.0, 15%(*v*/*v*) alcohol, and 200 mM MgCl_2_. (**A**–**C**) TEM images of the ^P156H^AfFer assemblies. (**D**) Real map of the inverted FFT from (**C**). The protein concentration was 2.0 µM. (**E**) 2D SAXS pattern of the ^P156H^AfFer assemblies. (**F**) Radially averaged 1D SAXS data of the ferritin assemblies. The simulated diffraction pattern is shown in blue.

To investigate the assembly behavior of ^P156H^AfFer in solution, firstly we analyzed the feasibility of ^P156H^AfFer performing BCT assembly with the crystallization conditions of native AfFer as a guide. Upon screening the solution conditions, similar platelet-like crystals were formed with ^P156H^AfFer at the low concentration (2.0 µM), which is six-fold lower than that required for the growth of above crystals, in a solution containing 10–20% alcohol and 200 mM MgCl_2_ at pH 8.0 (Figure S5), suggestive of a self-assembly process. TEM analyses revealed that the ^P156H^AfFer nanocages assembled into highly ordered architectures through symmetrical equivalent interactions (Figure 2A–C). On the basis of magnification of Figure 2C, the corresponding fast Fourier transform (FFT) image was recorded, and the real map from the inverted FFT provided an excellent view of the nanocage arrays, namely a ferritin molecule arranged in the center of four ferritin nanocages, resembling a BCT lattice (Figure 2D). To determine the superlattice structure and unit cell parameters in solution, small-angle X-ray scattering (SAXS) was performed to further evaluate the packing pattern of the ferritin assemblies [24,32]. The 2D SAXS data showed several distinct central diffraction rings, suggesting that a long periodic structure exists in the tested samples (Figure 2E) [18]. On the basis of the peak positions of the 1D SAXS curves, the unit cell parameters were derived as a = b = 11.98 nm and c = 20.74 nm, confirming that the protein nanocages are assembled in a BCT pattern (Figure 2F and Figure S6).

**Figure 3:**
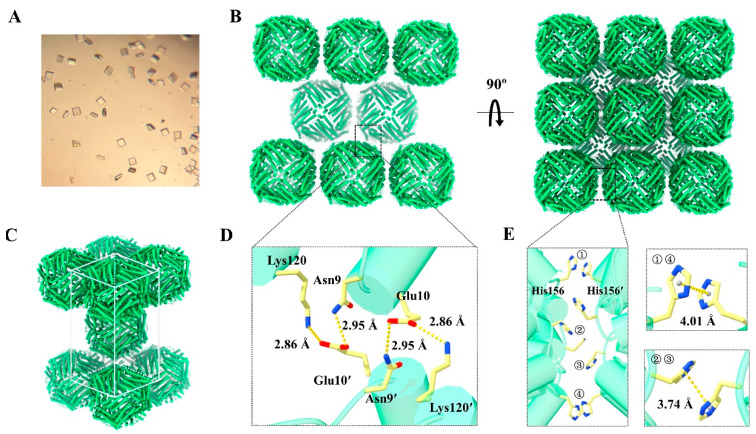
Body-centered tetragonal crystal structure of ^P156H^AfFer (PDB ID: 8IQY). (**A**) Optical microscope image of the crystal. (**B**) Side and top views of the 3D array in the crystal structure. (**C**) Tetragonal lattice. (**D**) Close-up view of the electrostatic interactions between two adjacent ^P156H^AfFer molecules along *C*_3_ interface. (**E**) Close-up view of His-mediated π–π interactions between two adjacent ^P156H^AfFer molecules along the *C*_4_ interface.

To elucidate the protein assembly behavior at the atomic level, we obtained ^P156H^AfFer flaky crystals (Figure 3A) and solved its crystal structure at a 2.10 Å resolution (Table S4). As expected, the ^P156H^AfFer nanocages were arranged in a BCT fashion in the crystals (Figure 3B–D). The designed His motifs of ^P156H^AfFer form four pairs of π–π stacking forces in a face-to-face configuration along at the *C*_4_ interfaces [33], whereas the inherent Pro156 residues in native AfFer stabilize the BCT arrangement through weak van der Waals forces. The crystal structure revealed that the imidazole ring distance of the pairing His–His is 4.01 Å or 3.74 Å (Figure 3E). Collectively, these findings demonstrate that ^P156H^AfFer nanocages have the ability to self-assemble into a BCT lattice in both solution and crystals through the designed His-mediated π–π stacking interactions at the *C*_4_ interfaces.

### 3.4. Construction and Characterization of 3D Protein Nanocage Superlattices with a SC Structure

Given that noncovalent interactions such as electrostatic interaction and π–π stacking are susceptible to pH and ionic strength, the assembly behavior could be regulated by adjusting the solution conditions. At 2.0 µM, the ^P156H^AfFer solution remained clear under different experimental conditions (pH from 7.0 to 10.0; the concentration of NaCl from 0 to 2000 mM). However, upon increasing the concentration of protein to 4.0 µM, the ^P156H^AfFer solution became cloudy. TEM analyses revealed that these ferritin molecules self-assemble into two kinds of protein arrays (Figure S7A): one is similar to a SC array, and the other is similar to the BCT array observed in crystals (Figure S7B), suggesting that ^P156H^AfFer is able to form two 3D arrays in solution simultaneously under the present conditions.

**Figure 4:**
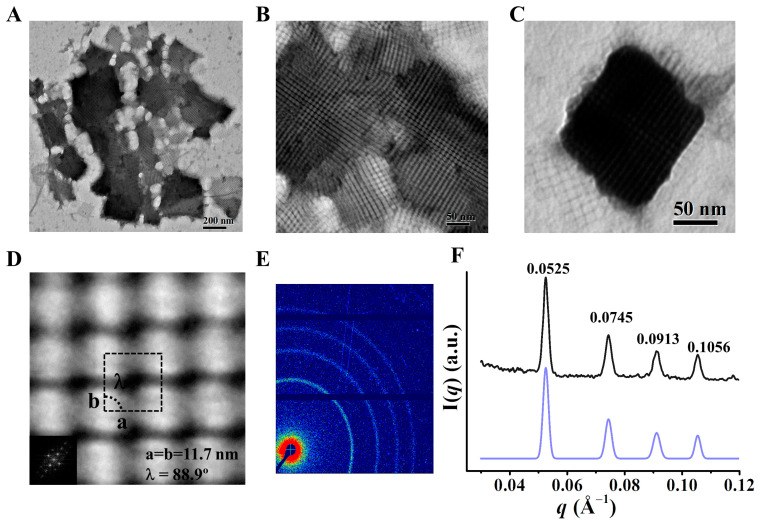
Characterization of the ^P156H^AfFer assemblies at pH 8.0 and 200 µM NiCl_2_. (**A**–**C**) TEM images of the ^P156H^AfFer assemblies. (**D**) Real map from the inverted FFT of (**B**). The protein concentration was 2.0 µM. (**E**) 2D SAXS pattern of the ^P156H^AfFer assemblies. (**F**) Radially averaged 1D SAXS data of the ferritin assemblies. Simulated diffraction pattern is shown in blue.

To induce the ^P156H^AfFer molecules to assemble into protein arrays with single SC lattices rather than the above observed mixture, we screened the solution conditions using different salt ions with different concentrations at pH 8.0. It was found that upon the addition of Ni^2+^ ions (100–400 equiv. of ferritin) to the protein solution (2.0 µM), the ^P156H^AfFer molecules form only one kind of highly ordered assembly in solution, as revealed by means of TEM (Figure 4A–C), whereas the wild-type AfFer nanocages remain almost monodispersed under the same conditions (Figure S8), demonstrating that His156 is responsible for the formation of the ordered protein assemblies. Afterwards, FFT based on TEM was performed to obtain a superior map of the ferritin array, which illustrates the following unit cell parameters: a = b = 11.7 nm, and λ = 88.9°, where a and b refer to the center-to-center distance between adjacent nanocages in columns or rows, respectively (Figure 4D). The value of 11.7 nm is consistent with the ferritin’s outer diameter of 12 nm, and the angle of 88.9° between the base vectors of the unit cell is close to a right angle of 90°, suggesting that the ^P156H^AfFer nanocages form SC superlattices. The resultant protein assemblies were further analyzed via SAXS to determine the arrangements and parameters of the superstructure. The samples gave rise to centric diffraction rings in the 2D SAXS, indicating a high long-range order and large domain size of the superlattice (Figure 4E,F). The identified *q* values from the azimuthally integrated curve stand at 0.0525, 0.0745, 0.0913, and 0.1056 Å^−1^, which correspond to the Bragg reflections of the SC lattice ((*hkl*) = (100), (110), (111), (200), *q*^n^/q* = 1: $\sqrt{2}$: $\sqrt{3}$: $\sqrt{4}$) [14]. The lattice constant determined from the data is a = 11.90 nm, corresponding to the interplanar distance of the cubic crystal system, which is nearly identical to the exterior diameter (~12 nm) of the ferritin nanocage. These results confirmed that the designed ^P156H^AfFer nanocages can self-assemble into pure SC arrays under the current conditions (Figure S9).

**Figure 5:**
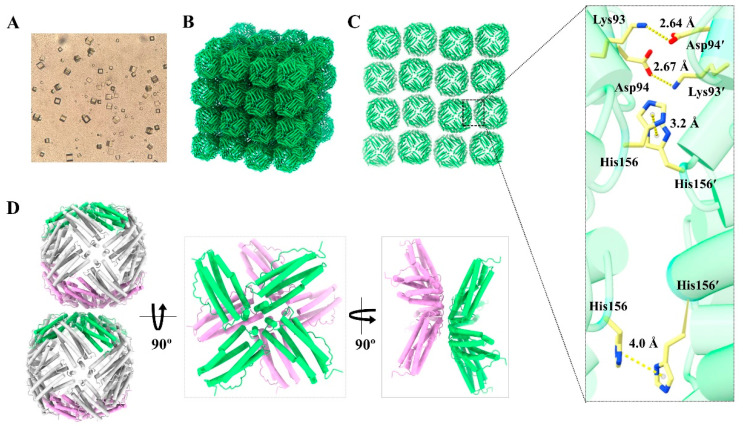
Simple cubic crystal structure of ^P156H^AfFer (PDB ID: 8IQX). (**A**) Optical microscope image of the crystal. (**B**) The SC array in the crystal structure. (**C**) Close-up view of the interfacial interactions between two adjacent ^P156H^AfFer molecules, including electrostatic and π–π stacking interactions. (**D**) Stacking pattern of two adjacent ^P156H^AfFer molecules along the *C*_4_ interface.

To elucidate the detailed structure of the 3D protein arrays, the high-quality cubic crystals of ^P156H^AfFer were obtained, and subsequently, its crystal structure was solved at a resolution of 2.50 Å (Figure 5A and Table S4). The space group is *I23*, with unit-cell parameters of a = b = c = 229.16 Å and α = β = γ, indicating that the SC lattice was yielded by ^P156H^AfFer (Figure 5B). As expected, the introduced histidine residues formed two pairs of π–π interactions between two neighboring ^P156H^AfFer molecules (Figure 5C) [33]. Additionally, two pairs of electrostatic attractions were formed by Lys93 and Asp94’ between adjacent ferritin molecules. Although such electrostatic attraction is also involved in the formation of the SC superlattices, the designed His-involved π–π interactions play a dominant role because wild-type AfFer cannot assemble into such SC superlattices. Although the adjacent ^P156H^AfFer cages are not aligned along the *C*_4_ rotation axes perfectly, the dislocation angles and distances are almost negligible relative to the entire cage-like structure of ferritin, and thus, they do not affect the overall SC structure (Figure 5D). The coordination of the nickel ions was not observed in the crystal (PDB ID: 8IQX). Agreeing with this observation, the same SC lattice (PDB ID: 8IQW) was obtained under other conditions without nickel ions: 100 mM imidazole pH 7.5, 35% MPD, and 200 mM MgCl_2_ (Table S4 and Figure S10). These results suggest that Ni^2+^ is able to induce the protein assembly into an SC lattice without the formation of coordination bonds with the designed His residues in the above protein assemblies. Interestingly, SC superlattices cannot be generated upon replacing Ni^2+^ with other ions such as Mg^2+^ and Ca^2+^, suggesting that the formation of the SC superlattices exhibits certain selectivity for Ni^2+^. The detailed mechanism of the Ni^2+^-induced protein assemblies is under investigation.

As a further example of the general use of our design strategy, another AfFer mutant named ^P156F^AfFer, where Pro156 was mutated into Phe156, was produced, which can also assemble into both BCT and SC superlattices. On the one hand, ^P156F^AfFer nanocages likewise can arrange into BCT lattices, as demonstrated by SAXS and X-ray diffraction (Figure S11A,C–E, PDB ID: 8IR0). On the other hand, ^P156F^AfFer molecules have the ability to pack into SC lattices upon adjusting the solution conditions (pH 7.0–9.0, 1.0 M–2.0 M NaCl), as demonstrated by SAXS data and X-ray diffraction (Figure S11B,F–H, PDB ID: 8IQZ). Differently, two adjacent ^P156F^AfFer molecules are perfectly aligned along the *C*_4_ rotation axes in the SC crystals. It is worth noting that the contact forces between adjacent protein molecules should only be composed of π–π attraction of Phe–Phe, as revealed by X-ray crystallography (Figure S11G and Table S5). Thus, if only adjusting the pH and NaCl concentration in the solution, ^P156H^AfFer forms two kinds of assemblies (Figure S7), while ^P156F^AfFer only forms SC assemblies (Figure S11B). Additionally, in their SC crystals, the *C*_4_ rotation axes of adjacent ^P156H^AfFer cages are not aligned perfectly, while the ^P156F^AfFer molecules are perfectly aligned. We believe that such a difference in protein assembly between ^P156F^AfFer and ^P156H^AfFer is most likely derived from the stronger π–π interactions of Phe–Phe pairs as compared to their analogues.

### 3.5. Reversible Assembly of BCT and SC Lattices

Protein assemblies mediated by non-covalent interactions have the ability to respond to external stimuli by changing their structure or assembly state. Since the BCT and SC lattices are constructed by the same nanocage through noncovalent interactions, they could interconvert with each other. To confirm this idea, we took ^P156H^AfFer as an example to explore the reversible assembly and disassembly characteristics. After the alcohol and salt ions involved in the formation of the assemblies were removed via dialysis, the protein solution regains pellucid (Figure S12A,B), indicative of a reversible process. Indeed, TEM analyses confirmed the disappearance of the previously observed two kinds of highly ordered assemblies, respectively, and instead, dispersed cage-like protein molecules were visualized (Figure S12C,D). These findings demonstrate that ^P156H^AfFer BCT and SC lattices can interconvert with each other, modulated by the solution conditions through ^P156H^AfFer nanocages as intermediates.

## 4. Conclusions

In this study, we have prepared starfish ferritin and resolved its crystal structure for the first time. It was found that AfFer is arranged in a BCT structure in the crystal. Inspired by the crystal structure of the AfFer nanocage, we established a facile, effective strategy by which two different kinds of 3D protein nanocage arrays can be constructed from identical protein building blocks. The method based on a single mutation of the amino acid residues near the protein interfaces is simple. The above two protein nanocage assemblies were confirmed by SAXS and X-ray crystallography at an atomic level. Notably, reversible conversion between the two different 3D arrays through discrete protein nanocages can be achieved. This study establishes a strategy for constructing diverse protein superlattices via the protein interface redesign of the same building blocks, which can be, theoretically, applied to other protein building blocks with similar symmetry. These 3D protein cage arrays provide three kinds of spaces: two sizes of interglobular lattice cavities as well as the inherent inner cavity of the protein nanocage, which can accommodate a wide range of guest molecules, such as metal nanoparticles, active small molecules, functional peptides, enzymes, etc., and thus they possess the potential for applications in food delivery, catalysis, detection, prevention and treatment, or other fields. Moreover, the interconversion of these superlattices would offer several advantages: (1) the spatial variation between the nanocages accommodates guest molecules of different sizes; (2) the physicochemical properties of the 3D lattice as well as of the encapsulated cargo vary with the arrangement; and (3) controlling the release of interglobular embedded molecules while maintaining a regular arrangement of cargoes in the cavity.

## Data Availability

Data are contained within the article and the Supplementary Materials.

## Supplementary Materials

The following supporting information can be downloaded at https://www.mdpi.com/article/10.3390/foods12213903/s1, Table S1: Primer used for the mutagenesis of AfFer; Table S2: Crystallization conditions for each crystal; Tables S3–S5: Crystallographic data collection and refinement statistics for AfFer, ^P156H^AfFer, ^P156F^AfFer; Figure S1: The subunit–subunit interfaces of ferritin, including the *C*_2_, *C*_3_, *C*_4_ and *C*_3_–*C*_4_ interfaces; Figure S2: SDS-PAGE and Native-PAGE analyses of AfFer; Figure S3: The three *C*_4_ axes of the AfFer nanocage and side chain of Pro156; Figure S4: SDS-PAGE and Native-PAGE analyses of the ^P156H^AfFer and ^P156F^AfFer variants; Figure S5: Optical microscope image of the ^P156H^AfFer crystals at pH 8.0, 15% (*v*/*v*) alcohol, and 200 mM MgCl_2_; Figure S6: Structural model of the body-centered tetragonal lattice; Figure S7: Characterization of the ^P156H^AfFer assemblies at pH 8.0 and 0.2 M NaCl; Figure S8: TEM images of AfFer at pH 8.0 and 200 µM NiCl_2_; Figure S9: Structural model of the simple cubic lattice; Figure S10: Crystal structure of ^P156H^AfFer at 100 mM imidazole pH 7.5, 35% MPD, and 200 mM MgCl_2_; Figure S11: SAXS and X-ray diffraction characterization of ^P156F^AfFer; Figure S12: Reversible self-assembly of the ^P156H^AfFer superlattices.

## Accession Codes

The crystal structures are deposited in the Protein Data Bank under the accession PDB IDs: 8IQV (AfFer), 8IQX and 8IQW (^P156H^AfFer-SC), 8IQY (^P156H^AfFer-BCT), 8IQZ (^P156F^AfFer-SC), and 8IR0 (^P156F^AfFer-BCT).

## References

[1] Zhang N., Yu X., Xie J., Xu H. (2021). New insights into the role of ferritin in iron homeostasis and neurodegenerative diseases. Mol. Neurobiol..

[2] Ashraf A., Jeandriens J., Parkes H.G., So P.-W. (2020). Iron dyshomeostasis, lipid peroxidation and perturbed expression of cystine/glutamate antiporter in alzheimer’s disease: Evidence of ferroptosis. Redox Biol..

[3] Chen H., Tan X., Han X., Ma L., Dai H., Fu Y., Zhang Y. (2022). Ferritin nanocage based delivery vehicles: From single-, co- to compartmentalized- encapsulation of bioactive or nutraceutical compounds. Biotechnol. Adv..

[4] Zhang C., Zhang X., Zhao G. (2020). Ferritin nanocage: A versatile nanocarrier utilized in the field of food, nutrition, and medicine. Nanomaterials.

[5] Lee N.K., Cho S., Kim I.-S. (2022). Ferritin-a multifaceted protein scaffold for biotherapeutics. Exp. Mol. Med..

[6] Luo Q., Hou C., Bai Y., Wang R., Liu J. (2016). Protein assembly: Versatile approaches to construct highly ordered nanostructures. Chem. Rev..

[7] Uchida M., Klem M.T., Allen M., Suci P., Flenniken M., Gillitzer E., Varpness Z., Liepold L.O., Young M., Douglas T. (2007). Biological containers: Protein cages as multifunctional nanoplatforms. Adv. Mater..

[8] Zhang J., Wang Q., Liu B., Li D., Zhang H., Wang P., Liu J., Hou G., Li X., Yuan Y. (2022). The kinetic mechanism of cations induced protein nanotubes self-assembly and their application as delivery system. Biomaterials.

[9] Abe S., Pham T.T., Negishi H., Yamashita K., Hirata K., Ueno T. (2021). Design of an in-cell protein crystal for the environmentally responsive construction of a supramolecular filament. Angew. Chem. Int. Ed..

[10] Shen H., Fallas J.A., Lynch E., Sheffler W., Parry B., Jannetty N., Decarreau J., Wagenbach M., Vicente J.J., Chen J. (2018). De novo design of self-assembling helical protein filaments. Science.

[11] Zhang S., Alberstein R.G., De Yoreo J.J., Tezcan F.A. (2020). Assembly of a patchy protein into variable 2d lattices via tunable multiscale interactions. Nat. Commun..

[12] Gonen S., DiMaio F., Gonen T., Baker D. (2015). Design of ordered two-dimensional arrays mediated by noncovalent protein-protein interfaces. Science.

[13] McCoy K., Uchida M., Lee B., Douglas T. (2018). Templated assembly of a functional ordered protein macromolecular framework from p22 virus-like particles. ACS Nano.

[14] Zhou K., Zang J., Chen H., Wang W., Wang H., Zhao G. (2018). On-axis alignment of protein nanocage assemblies from 2d to 3d through the aromatic stacking interactions of amino acid residues. ACS Nano.

[15] Zhu J., Avakyan N., Kakkis A., Hoffnagle A.M., Han K., Li Y., Zhang Z., Choi T.S., Na Y., Yu C.J. (2021). Protein assembly by design. Chem. Rev..

[16] Lv C., Zhang X., Liu Y., Zhang T., Chen H., Zang J., Zheng B., Zhao G. (2021). Redesign of protein nanocages: The way from 0d, 1d, 2d to 3d assembly. Chem. Soc. Rev..

[17] Chakraborti S., Korpi A., Kumar M., Stępień P., Kostiainen M.A., Heddle J.G. (2019). Three-dimensional protein cage array capable of active enzyme capture and artificial chaperone activity. Nano Lett..

[18] Künzle M., Eckert T., Beck T. (2016). Binary protein crystals for the assembly of inorganic nanoparticle superlattices. J. Am. Chem. Soc..

[19] Tian Y., Lhermitte J.R., Bai L., Vo T., Xin H.L., Li H., Li R., Fukuto M., Yager K.G., Kahn J.S. (2020). Ordered three-dimensional nanomaterials using DNA-prescribed and valence-controlled material voxels. Nat. Mater..

[20] Laramy C.R., O’Brien M.N., Mirkin C.A. (2019). Crystal engineering with DNA. Nat. Rev. Mater..

[21] Zeng R., Zhang X., Zhang T., Lv C., Zang J., Zhao G. (2022). Directed compartmentalization of mg^2+^ and carbonic anhydrase by designed three-dimensional protein crystalline frameworks for efficient conversion of CO_2_ into nanoparticles. ACS Sustain. Chem. Eng..

[22] Zhang X., Zeng R., Zhang T., Lv C., Zang J., Zhao G. (2022). Spatiotemporal control over 3d protein nanocage superlattices for the hierarchical encapsulation and release of different cargo molecules. J. Mater. Chem. B.

[23] Masuda T., Zang J., Zhao G., Mikami B. (2018). The first crystal structure of crustacean ferritin that is a hybrid type of h and l ferritin. Protein Sci..

[24] Bailey J.B., Zhang L., Chiong J.A., Ahn S., Tezcan F.A. (2017). Synthetic modularity of protein-metal-organic frameworks. J. Am. Chem. Soc..

[25] Sontz P.A., Bailey J.B., Ahn S., Tezcan F.A. (2015). A metal organic framework with spherical protein nodes: Rational chemical design of 3d protein crystals. J. Am. Chem. Soc..

[26] Beck G., Ellis T.W., Habicht G.S., Schluter S.F., Marchalonis J.J. (2002). Evolution of the acute phase response: Iron release by echinoderm (*Asterias forbesi*) coelomocytes, and cloning of an echinoderm ferritin molecule. Dev. Comp. Immunol..

[27] Zhang C., Liu Y., Zhang T., Lv C., Zang J., Zhao G. (2022). Structural comparison between the DNA-protective ability of scallop and shrimp ferritin from iron-induced oxidative damage. Food Chem..

[28] Minor W., Cymborowski M., Otwinowski Z., Chruszcz M. (2006). Hkl-3000: The integration of data reduction and structure solution—From diffraction images to an initial model in minutes. Acta Crystallogr. Sect. D.

[29] Afonine P.V., Grosse-Kunstleve R.W., Echols N., Headd J.J., Moriarty N.W., Mustyakimov M., Terwilliger T.C., Urzhumtsev A., Zwart P.H., Adams P.D. (2012). Towards automated crystallographic structure refinement with phenix.Refine. Acta Crystallogr. Sect. D.

[30] Emsley P., Cowtan K. (2004). Coot: Model-building tools for molecular graphics. Acta Crystallogr. Sect. D.

[31] Pettersen E.F., Goddard T.D., Huang C.C., Meng E.C., Couch G.S., Croll T.I., Morris J.H., Ferrin T.E. (2021). Ucsf chimerax: Structure visualization for researchers, educators, and developers. Protein Sci..

[32] Li T., Senesi A.J., Lee B. (2016). Small angle X-ray scattering for nanoparticle research. Chem. Rev..

[33] Tan X., Chen H., Gu C., Zang J., Zhang T., Wang H., Zhao G. (2020). Converting histidine-induced 3D protein arrays in crystals into their 3D analogues in solution by metal coordination cross-linking. Commun. Chem..

