# Supplementary material for: Preparation and Unique Three-Dimensional Self-Assembly Property of Starfish Ferritin
Source: Foods. 2023 Oct 25;12(21):3903. doi: 10.3390/foods12213903 (PMC10647799; doi:10.3390/foods12213903)
Supplement: Supplementary file 1 [file foods-12-03903-s001.zip › foods-2680943-supplementary.pdf]

Supporting Information

for

Preparation and Unique Three-Dimensional Self-Assembly Property of Starfish Ferritin

Chenxi Zhang, Xuemin Chen, Bo Liu, Jiachen Zang, Tuo Zhang and Guanghua Zhao *

College of Food Science & Nutritional Engineering, China Agricultural University, Beijing 100083, China; zhangchenxi9556@163.com (C.Z.); s20213061072@cau.edu.cn (X.C.); s20193060993@cau.edu.cn (B.L.); zangjiachen@cau.edu.cn (J.Z.); zhangtuo@cau.edu.cn (T.Z.)

* Correspondence: gzhao@cau.edu.cn

Results and Discussion

Supplementary Table

Table S1. Primer used for the mutagenesis of AfFer.

Mutation	Primer
^{P156H}AfFer	5’- CAGCTGACCCGTGTGGGTCACGGTCTGGGCGAATATACC -3’
	5’- GGTATATTCGCCCAGACCGTGACCCACACGGGTCAGCTG -3’
^{P156F}AfFer	5’- CAGCTGACCCGTGTGGGTTTCGGTCTGGGCGAATATACC -3’
	5’- GGTATATTCGCCCAGACCGAAACCCACACGGGTCAGCTG -3’

Table S2. Crystallization conditions for each crystal.

Proteins	Crystallization conditions	pH
AfFer	100 mM imidazole, 15%(v/v) ethanol, 200 mM $MgCl_2$	8.0
^{P156H}AfFer-SC①	100 mM imidazole, 15%(v/v) ethanol, 200 mM $MgCl_2$, 2.0 mM $NiCl_2$	8.0
^{P156H}AfFer-SC②	100 mM imidazole, 35% MPD, 200 mM $MgCl_2$	7.5
^{P156H}AfFer-BCT	100 mM imidazole, 15%(v/v) ethanol, 200 mM $MgCl_2$	8.0
^{P156F}AfFer-SC	100 mM Tris, 1.0 M NaCl	9.0
^{P156F}AfFer-BCT	100 mM MES, 20%(v/v) 1,4-butanediol, 200 mM Li_2SO_4	6.0

Table S3. Crystallographic data collection and refinement statistics for AfFer.

**Parameters**	**AfFer**
**Data collection**	
Beamline	SSRF BL17U1
Wavelength (Å)	1.2915
Space group	*I* 4 2 2
Unit cell	116.52 116.52 205.62<br>90 90 90
Resolution (Å)	43.61-1.91
Completeness (%)	99.04
$CC_{1/2}$	0.997
Unique reflections	54843
Measured reflections	673257
**Refinement**	
R_{work}/R_{free}	0.219 / 0.248
Wilson B-factor (Å^2)	22.64
B-factors (Å^2)	25.31
Rmsd bond lengths (Å)	0.008
Rmsd bond angles (°)	0.827
Ramachandran plot (%)	
Favored (%)	99.19
Outliers (%)	0

Table S4. Data collection and refinement statistics for ^{P156H}AfFer.

**Parameters**	^{P156H}**AfFer**-SC①	^{P156H}**AfFer**-SC②	^{P156H}**AfFer**-BCT
**Data collection**			
Beamline	SSRF BL18U		
Wavelength (Å)	0.9792		
Space group	*I* 2 3	*I 2 3*	*I* 4 2 2
Unit cell	229.16 229.16 229.16<br>90 90 90	228.47 228.47 228.47<br>90 90 90	116.93 116.93 206.54<br>90 90 90
Resolution (Å)	28.64-2.50	30.0-2.50	27.56-2.10
Redundancy	18.5	18.2	13.0
Completeness (%)	99.84	99.88	99.77
$I/\sigma I$	2.2	2.6	2.7
R_{merge}	0.161	0.196	0.141
$CC_{1/2}$	0.929	0.917	0.943
Unique reflections	68784	67917	42226
Measured reflections	1269561	1233504	549920
**Refinement**			
R_{work}/R_{free}	0.2133 / 0.2563	0.2343 / 0.2921	0.165 / 0.183
Wilson B-factor (Å^2)	28.69	26.12	23.49
B-factors (Å^2)	35.29	29.16	25.07
Rmsd bond lengths (Å)	0.009	0.008	0.007
Rmsd bond angles (°)	1.051	0.985	0.788
Ramachandran plot (%)			
Favored (%)	97.80	97.93	99.39
Outliers (%)	0	0	0

Table S5. Data collection and refinement statistics for ^{P156F}AfFer.

**Parameters**	$^{\mathbf{P156F}}$**AfFer**-SC	$^{\mathbf{P156F}}$**AfFer**-BCT
**Data collection**		
Beamline	SSRF BL18U	
Wavelength (Å)	0.9792	
Space group	*P* 31 2 1	*I* 4 2 2
Unit cell	160.71 160.71 199.73<br>90 90 120	116.51 116.51 206.66<br>90 90 90
Resolution (Å)	28.55-4.19	29.65-2.89
Redundancy	5.4	12.7
Completeness (%)	99.15	99.71
$I/\sigma I$	2.7	2.0
R_{merge}	0.313	0.202
$CC_{1/2}$	0.743	0.972
Unique reflections	22303	16300
Measured reflections	119885	207058
**Refinement**		
R_{work}/R_{free}	0.214 / 0.267	0.185 /0.234
Wilson B-factor (Å^2)	79.48	45.11
B-factors (Å^2)	84.22	38.63
Rmsd bond lengths (Å)	0.003	0.011
Rmsd bond angles (°)	0.461	1.120
Ramachandran plot (%)		
Favored (%)	98.18	97.78
Outliers (%)	0	0

Supplementary Figures

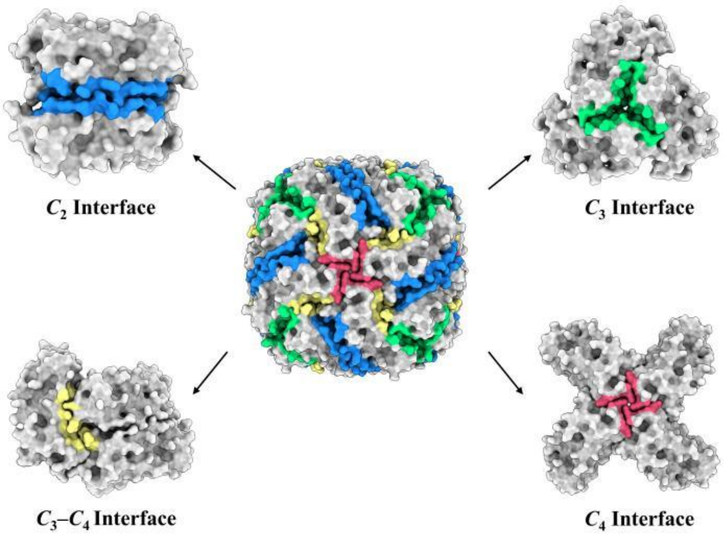

Figure S1. The subunit–subunit interfaces of ferritin, including the C_2, C_3, C_4 and C_3–C_4 interfaces.

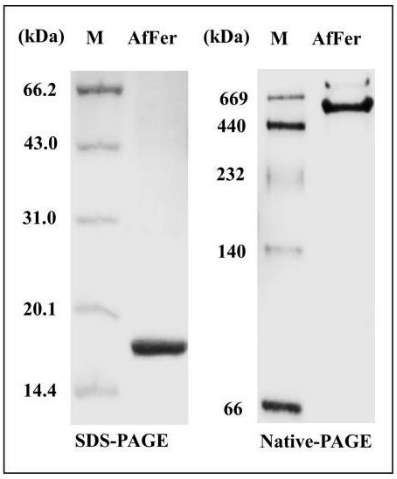

Figure S2. SDS-PAGE and Native-PAGE analyses of AfFer.

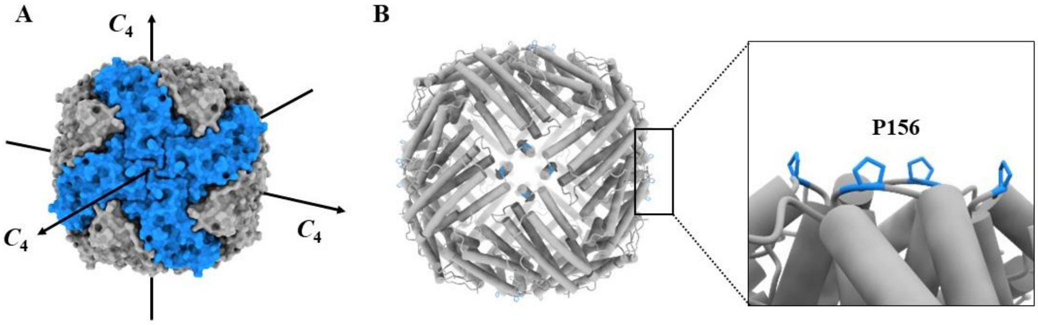

Figure S3. (A) The three C_4 axes of the AfFer nanocage are located along the X−Y−Z coordinate axes when the cavity center as the origin of the coordinates. (B) Side chain of Pro156 of AfFer.

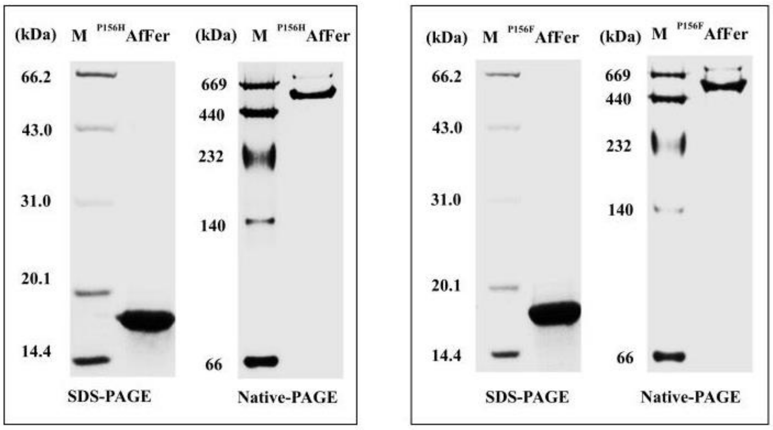

Figure S4. SDS-PAGE and Native-PAGE analyses of the ^{P156H}AfFer and ^{P156F}AfFer variants.

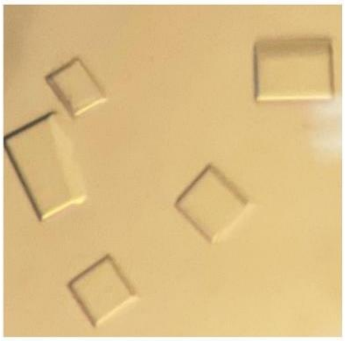

Figure S5. Optical microscope image of the ^{P156H}AfFer crystals at pH 8.0, 15%(v/v) alcohol, and 200 mM $MgCl_2$. The protein concentration was ~2.0 µM.

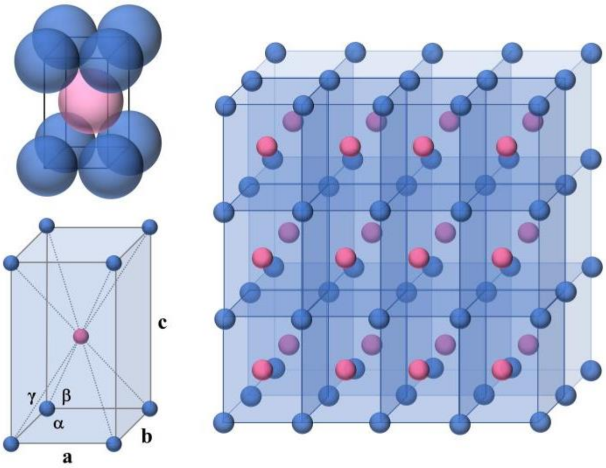

Figure S6. Structural model of the body-centered tetragonal lattice.

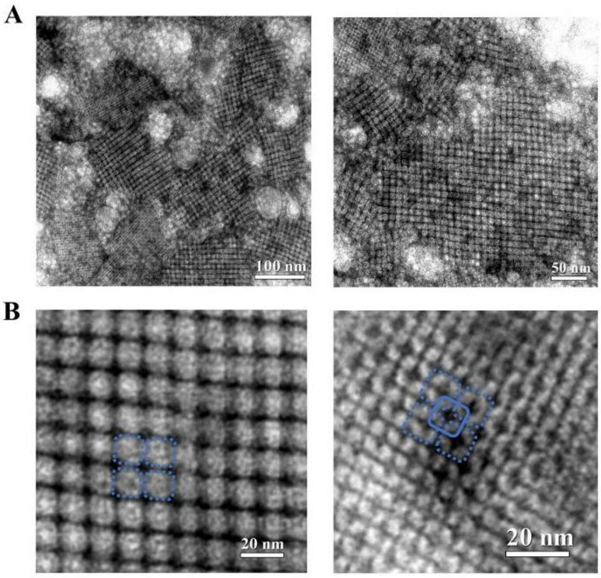

Figure S7. Characterization of the ^{P156H}AfFer assemblies at pH 8.0 and 0.2 M NaCl. (A) TEM images of the ^{P156H}AfFer assemblies. (B) Real map form inverted FFT of (A). Two different types of protein lattices were highlighted in blue. The protein concentration was 4.0 μM.

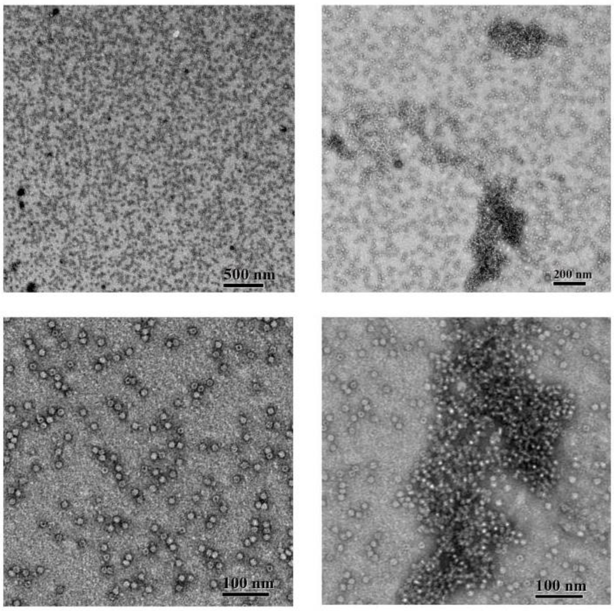

Figure S8. TEM images of AfFer at pH 8.0 and 200 µM $NiCl_2$. The protein concentration was 2.0 µM.

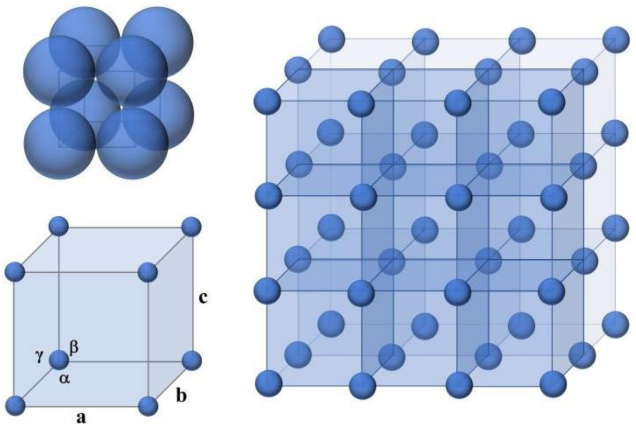

Figure S9. Structural model of the simple cubic lattice.

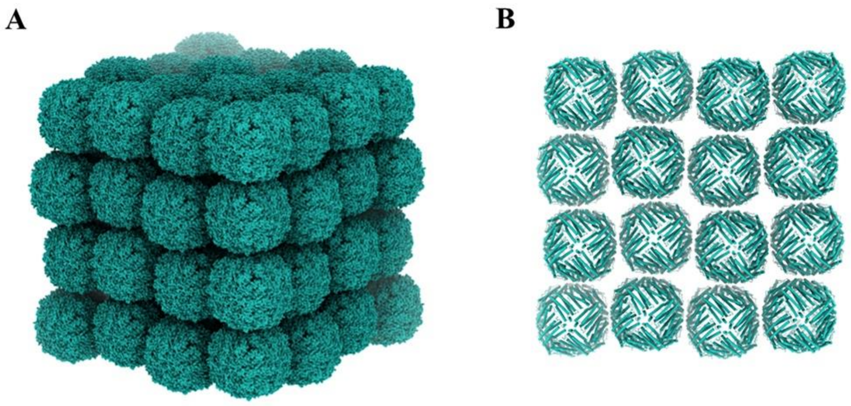

Figure S10. Crystal structure of ^{P156H}AfFer at 100 mM imidazole pH 7.5, 35% MPD, and 200 mM $MgCl_2$. (A) The SC array in the crystal structure. (B) Side view of the SC arrays in the crystal structure (PDB ID: 8IQW)

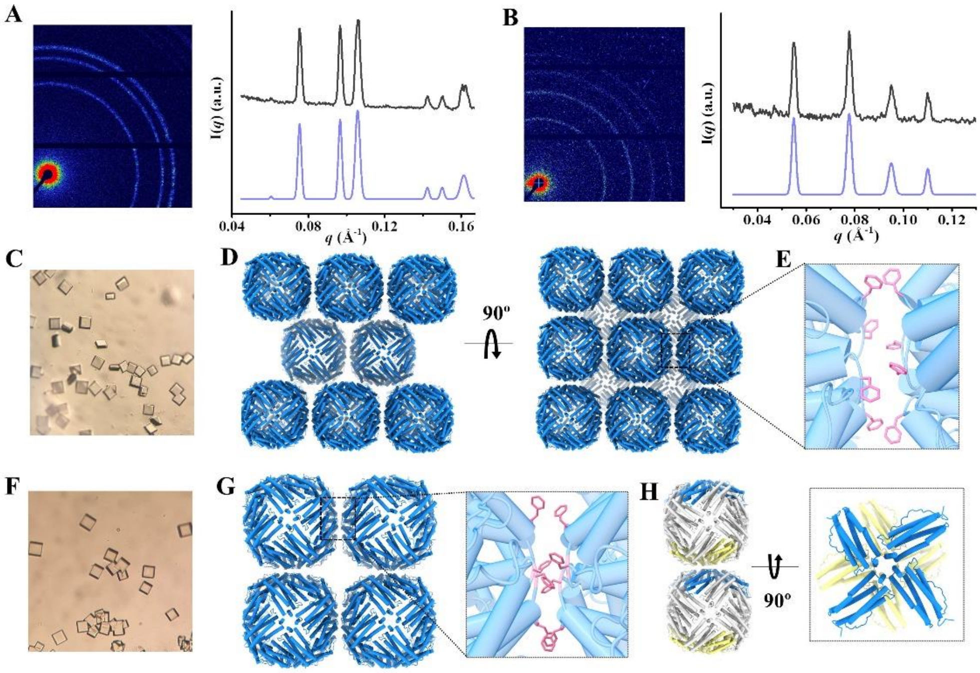

Figure S11. SAXS and X-ray diffraction characterization of ^{P156F}AfFer. 2D SAXS pattern and radially averaged 1D SAXS data for BCT (A) or SC assemblies (B) of ^{P156F}AfFer. Simulated diffraction pattern is shown in blue. (C) Optical microscope image of the BCT crystals. (D) Side and top views of the BCT arrays in the crystal structure (PDB ID: 8IR0). (E) Close-up view of the Phe156 at the C_4 interface between two adjacent ^{P156F}AfFer molecules in BCT crystal. (F) Optical microscope image of the SC crystals. (G) The SC lattice in the crystal and close-up view of the Phe156 at the C_4 interface between two adjacent ^{P156F}AfFer molecules (PDB ID: 8IQZ). (H) Stacking pattern of the C_4 interface between two adjacent ^{P156F}AfFer molecules.

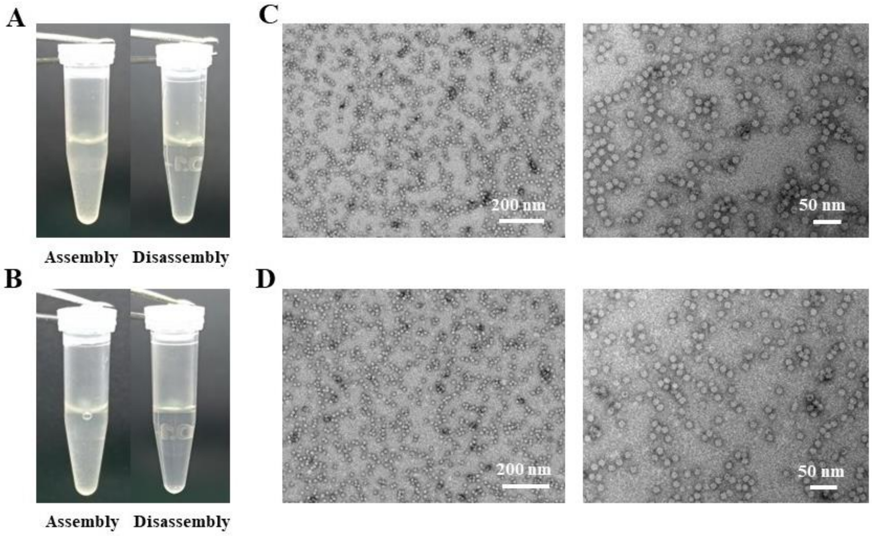

Figure S12. Reversible self-assembly of the ^{P156H}AfFer superlattices. Solution photographs of BCT (A) and SC (B) assemblies before and after dialysis. TEM characterization of BCT (C) and SC (D) assemblies after dialysis.
